# Monitoring Severe Slugging in Pipeline-Riser System Using Accelerometers for Application in Early Recognition

**DOI:** 10.3390/s19183930

**Published:** 2019-09-12

**Authors:** Sunah Jung, Haesang Yang, Kiheum Park, Yutaek Seo, Woojae Seong

**Affiliations:** 1 Department. of Naval Architecture and Ocean Engineering, Seoul National University, Seoul 151-744, Korea; sunj@snu.ac.kr (S.J.); coupon3@snu.ac.kr (H.Y.); rlgma16@snu.ac.kr (K.P.); yutaek.seo@snu.ac.kr (Y.S.); 2 Research Institute of Marine Systems Engineering, Seoul National University, Seoul 151-744, Korea

**Keywords:** severe slugging, multiphase flow, accelerometer, support vector machine, neural network

## Abstract

The use of accelerometer signals for early recognition of severe slugging is investigated in a pipeline-riser system conveying an air–water two-phase flow, where six accelerometers are installed from the bottom to the top of the riser. Twelve different environmental conditions are produced by changing water and gas superficial velocities, of which three conditions are stable states and the other conditions are related to severe slugging. For online recognition, simple parameters using statistics and linear prediction coefficients are employed to extract useful features. Binary classification to recognize stable flow and severe slugging is performed using a support vector machine and a neural network. In multiclass classification, the neural network is adopted to identify four flow patterns of stable state, two types of severe slugging, and an irregular transition state between severe slugging and dual-frequency severe slugging. The performance is compared and analyzed according to the signal length for three cases of sensor location: six accelerometers, one accelerometer at the riser base, and one accelerometer at the top of the riser.

## 1. Introduction

Pipeline-riser structures composed of a production well, subsea pipeline, and vertical riser are generally used in offshore oil and gas fields. Mixtures of liquids, gases, and solid components are extracted from a reservoir, transported through a horizontal subsea pipeline of several kilometers, and raised to the topside via a vertical riser near the platform. In a pipeline-riser system, a more complicated multiphase flow can be developed than the flow from a simple horizontal or vertical pipe. In particular, a typical severe slugging can be generated in an undesired cycle with unstable blowout, causing large fluctuations in pressure that lead to structural damage and reduce the production rate [1,2]. In detail, the severe slugging can generate liquid overflow, high pressure in the separators, overload on the gas compressors, extra fatigue by repeated impact, increased corrosion, and low production [3,4,5,6]. In offshore pipeline facilities, stable operation is important for safety and economic benefits; therefore, unstable flow should be identified as quickly as possible.

Many studies have classified flow regimes using machine learning algorithms in simple horizontal, vertical pipe, or pipeline-riser systems [7,8,9,10,11,12,13,14]. Wu et al. [12] performed an experiment in a simple horizontal pipe using mixtures of mineral oil, air, and water to simulate oil-gas-water flow, and measured differential pressures. Features were extracted using the fractal dimension from denoised signals by the wavelet transform. A neural network (NN) was employed to classify stratified, intermittent, and annular flows. Trafalis [11] investigated flow recognition with a support vector machine (SVM) in a simple horizontal and a simple vertical pipe by applying gas and liquid superficial velocities and pipe diameter as features. Three regimes of bubble, intermittent, and annular flows were identified for the vertical flow, and four categories of annular, bubble, intermittent, and stratified flows were recognized for the horizontal flow. However, as superficial velocities are difficult to acquire in industrial pipelines, applying them to real industrial environments is not easy.

Meanwhile, in studies of the pipeline-riser systems, Goudinakis [8] obtained differential pressures in an S-shaped riser system. The normalized differential pressures were used as inputs of the NN, where the length of sample was 100 s. Four classes of bubble, oscillation (OSC), slug, and severe slugging 1 (SS1) were classified. Ye and Guo [13] also conducted an experiment in an S-shaped riser and acquired differential pressure signals of 20-min length. Least-squares SVM (LS-SVM) classifiers were trained with features of statistical parameters such as absolute mean, variance, skewness, and power spectral densities. SS1, severe slugging 2 (SS2), severe slugging transition, OSC, and stable flow were identified. Zou et al. [14] measured the differential pressure in a pipeline-riser system. For fast recognition, they computed the mean and range of differential pressures as feature vectors for the LS-SVM. They analyzed the results for various signal lengths and, in particular, the shortest signals of 6.8 s were tested by the classifier trained using 8.99 s signals. Four classes of severe slugging, OSC, stable like flow, and stable flow were recognized. In the case of a commonly used pressure gauge applied in the aforementioned studies, it is low-cost and usable at wide ranges of pressures and temperatures. However, it has some disadvantages: (1) a pressure tap can be blocked; (2) a pressure signal can be affected by either the water’s or gas’s velocity as well as by the flow regime [10]; (3) it is difficult to move into another location once it is installed.

In addition to the previously mentioned measuring instrument, other devices have been used to identify the flow regime. Applications of electrical impedance measurements for flow regime recognition have been implemented by Hernández et al. [15], Juliá et al. [16], and Mi et al. [9]. As the impedance sensor’s output is proportional to the void fraction, which is related to the flow pattern, its computational cost is relatively low [10]. However, it is only applicable to two-phase flow where the conductivities and dielectric coefficients of the gas and liquid are significantly different from each other [12]. Furthermore, it is sensitive to temperature and requires that the pipe should be composed of nonconducting material. Therefore, it may be difficult to operate in the industrial field.

The purpose of this study is to investigate severe slugging identification in a pipeline-riser system using accelerometer signals. Accelerometers are operated in a nonintrusive fashion and can be transferred to other locations after the initial installation, according to user’s desire or operational purpose. In addition, they can result in better classification performance, as the vibration characteristics are considerably different between stable flow and severe slugging, especially during liquid accumulation of the severe slugging process. In this study, a laboratory experiment for severe slugging monitoring in a pipeline-riser system using accelerometers is performed. Although the accelerometers are mounted in the air in the laboratory experiment, they could be applied in both the water and air, such as near the bottom of the riser or close to the topside in a real pipeline-riser system. For early recognition of the flow regime, relatively simple features characterizing flow-induced vibration (FIV) are presented based on statistical parameters and linear prediction coefficients (LPCs). The classification performance is compared and analyzed according to the signal length for different sensor selections using six accelerometers, one accelerometer at the riser base, and one accelerometer at the top of the riser. Two machine learning algorithms using the SVM and NN are applied to binary classification, and the NN is adopted for multiclass classification to recognize four classes: stable, SS1, and an irregular transition between severe slugging 3 (SS3) and dual-frequency severe slugging (DFSS).

This paper is organized as follows: Section 2 describes the background of severe slugging, including its processes and types. Section 3 presents the laboratory experiment in a pipeline-riser system under various environmental conditions of air and water superficial velocities, and presents the characteristics of accelerometer signals. In Section 4, a simple description of statistical parameters and LPCs as features for machine learning algorithms is given. In addition, binary and multiclass classification results using the SVM and NN are demonstrated. Section 5 summarizes this work and draws conclusions.

## 2. Description of Severe Slugging

**Figure 1:**
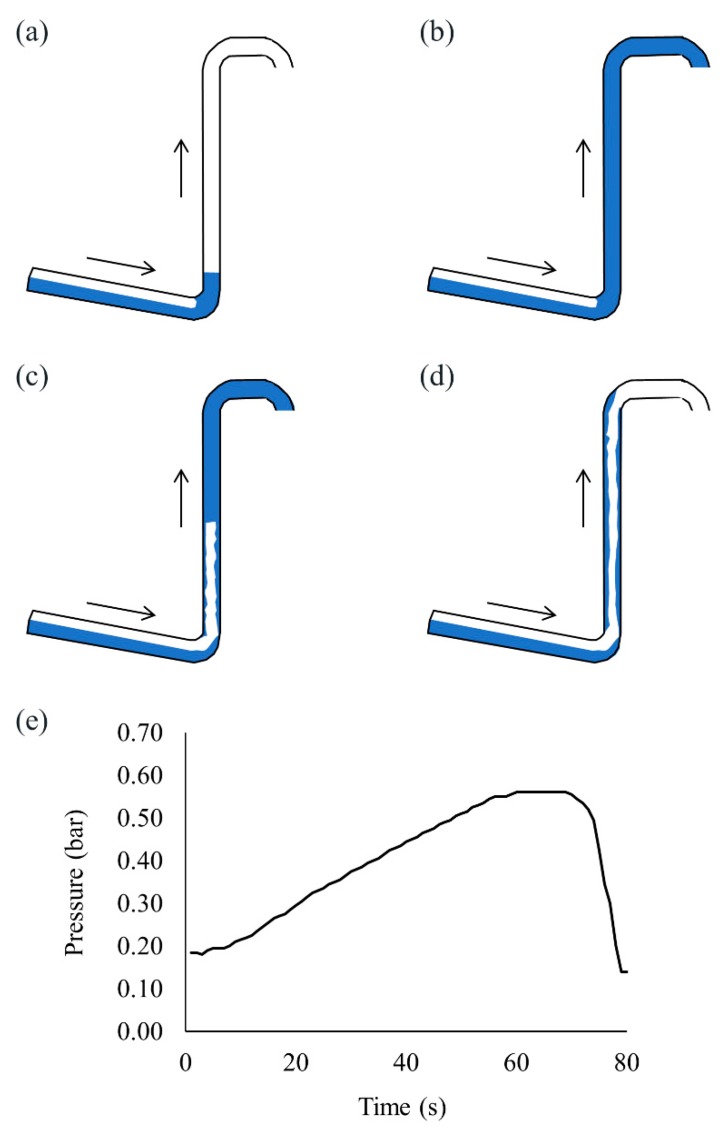
Process of severe slugging. (**a**) Slug formation; (**b**) slug movement into the separator; (**c**) blowout; (**d**) liquid fallback and (**e**) pressure at the riser base during one cycle of severe slugging.

Severe slugging is generally explained by the following four steps: (1) slug formation (2) slug movement into the separator (3) blowout and (4) liquid fallback [17,18]. Figure 1a–d present the process of severe slugging and Figure 1e shows the pressure at the bottom of the riser (*p_B_*) during one cycle of severe slugging, where the water and gas superficial velocities are $u_{SW}$ = 0.19 m/s and $u_{SG}$ = 0.59 m/s, respectively. During slug formation in Figure 1a, liquid transported from the pipeline starts to accumulate at the bottom of the riser and *p_B_* continuously increases for less than approximately 60 s as shown in Figure 1e, because the hydrostatic pressure increases by accumulated liquid in the riser. At the same time, the gas is blocked in the pipeline section and compressed. In the second stage of Figure 1b, the accumulated liquid reaches the top of the riser and moves into the separator while the blocked gas is continuously compressed, and *p_B_* increases to its maximum. When the pressure of the compressed gas surpasses the hydrostatic pressure of the accumulated liquid in the riser, the gas expands and blows out rapidly, as shown in Figure 1c, and *p_B_* starts to decrease. After the blowout, the remaining liquid in the riser falls back to the riser base as in Figure 1d and the slug formation repeats.

**Figure 2:**
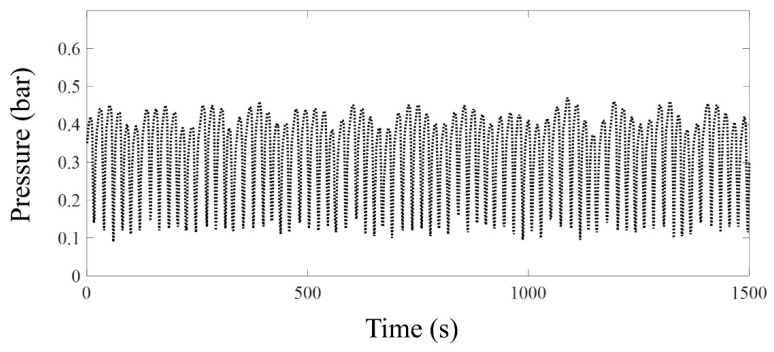
Typical pressure signal near the riser base of DFSS. The water and gas superficial velocities are $u_{SW}$ = 0.15 m/s and $u_{SG}$ = 0.99 m/s, respectively. The period of the high-frequency element is approximately 20 s, and that of low-frequency part is roughly 100 s.

In general, severe slugging can be classified into three types [19,20]: SS1, SS2, and SS3. SS1 and SS2 are similar, except for the length of the liquid slug. The liquid slug length of SS1 is longer than or equal to the height of the riser, whereas that of SS2 is shorter than the riser length. In case of SS2, the gas penetrates the riser before the liquid reaches the top of the riser, and as a result, the maximum *p_B_* is lesser than that of SS1. SS3, however, is quite different from SS1 and SS2. The gas continuously moves into the riser and creates transient slugs of different sizes. The liquid from the transient slugs falls into the bottom of the riser, accumulates again, and produces a long aerated liquid slug with small bubbles. Similarly, when the compressed gas overcomes the hydrostatic pressure of the stacked aerated liquid slugs, the gas blows out and the cycle repeats. In addition to these three types of severe slugging, Malekzadeh et al. [20] introduced another type of severe slugging, named DFSS. DFSS is related to SS3 and OSC, where OSC is characterized as cyclic pressure fluctuations without intense blowout [19]. With respect to DFSS, the liquid content is insufficient to generate SS3 and, at the same time, the gas flow rate is insufficient to maintain OSC. Accordingly, DFSS presents tendencies of both SS3 and OSC, resulting in two different frequencies. The high-frequency component is associated with fluctuations of both SS3 and OSC, because they have their own variations, and the low-frequency element is related to periodic transitions between SS3 and OSC. A typical example of the pressure *p_B_* for DFSS is depicted in Figure 2, as measured in our laboratory experiment at $u_{SW}$ = 0.15 m/s and $u_{SG}$ = 0.99 m/s. In Figure 2, the period of the high-frequency component is approximately 20 s, and that of the low-frequency element is approximately 100 s. In this study, the unstable flow regimes of SS1, and the irregular transition between DFSS and SS3 are investigated.

## 3. Experimental Apparatus and Procedure

**Figure 3:**
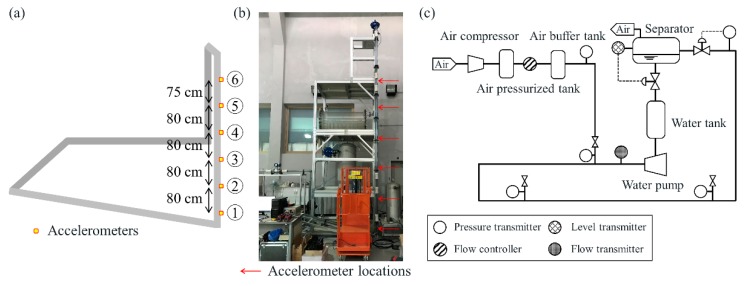
Experimental setup. (**a**) Location of accelerometer sensors; (**b**) photograph of the vertical riser, and (**c**) schematic diagram of the flow loop. The six sensor numbers are denoted as 1–6 in (**a**). The pipe is composed of PVC, and its inner diameter is 5.08 cm with a 5-mm thickness. The length of the inclined pipeline is 6.28 m at −15.9° to the horizontal and the height of the vertical riser is 5.6 m.

The experiment was conducted in the flow loop of the Subsea Engineering and Flow Assurance Laboratory at Seoul National University, as shown in Figure 3. The facility consists of a 6.28-m-long downward-inclined pipeline at −15.9° to the horizontal and a 5.6-m-high vertical riser. The pipe is composed of PVC and its inner diameter is 5.08 cm, with 5 mm thickness. The flow loop uses water and air as liquid and gas phases, respectively. The air is compressed to 8 bar by a compressor and stored in pressurized tank. A mass flow controller can control the air injection and an air buffer tank provides enough volume for air. The water is pumped from a water tank using a NETZSCH pump with maximum liquid flow rate 85.14 L/min, which is a constant flow pump producing same flow rate regardless of pressure fluctuation between the front and rear ends. At the top of the riser, a globe type valve is installed to control the flow rate and mitigate the severe slugging. Six accelerometers measuring out-of-plane accelerations were installed at the riser, as indicated by the square markers in Figure 3a and the red arrows in Figure 3b. For convenience, the accelerometers are numbered from 1 to 6 according to the location of the sensors, from the bottom to the top of the riser. Accelerometers 1, 3, 5, and 6 of PCB type 352A60 were installed at *h* = 0.27, 1.87, 3.47, and 4.22 m from the floor, respectively, and connected to a B&K NEXUS conditioning amplifier 2693. The sensitivity of PCB type 352A60 is 1.02 mV/ms^−2^ and its frequency range is from 5 Hz to 60 kHz. The other two accelerometers, 2 and 4, of B&K type 4384, mounted at *h* = 1.07 and 2.67 m, were connected to a B&K NEXUS conditioning amplifier 2692-C. The sensitivity of B&K type 4384 is 1 pC/ms^−2^ available between 0.1 Hz and 12.6 kHz. The vertical distance between each of the lower five accelerometers was 0.8 m, and the vertical distance between accelerometers 5 and 6 was 0.75 m. The length of accelerometer data was 10 s and the sampling rate was 50 kHz. In addition, five WIKA model A-10 pressure transmitters were installed as shown in Figure 3c, and the pressure signal was sampled every second. In this study, only pressure signal obtained from the bottom of the riser is investigated as a reference for the accelerometer data.

**Figure 4:**
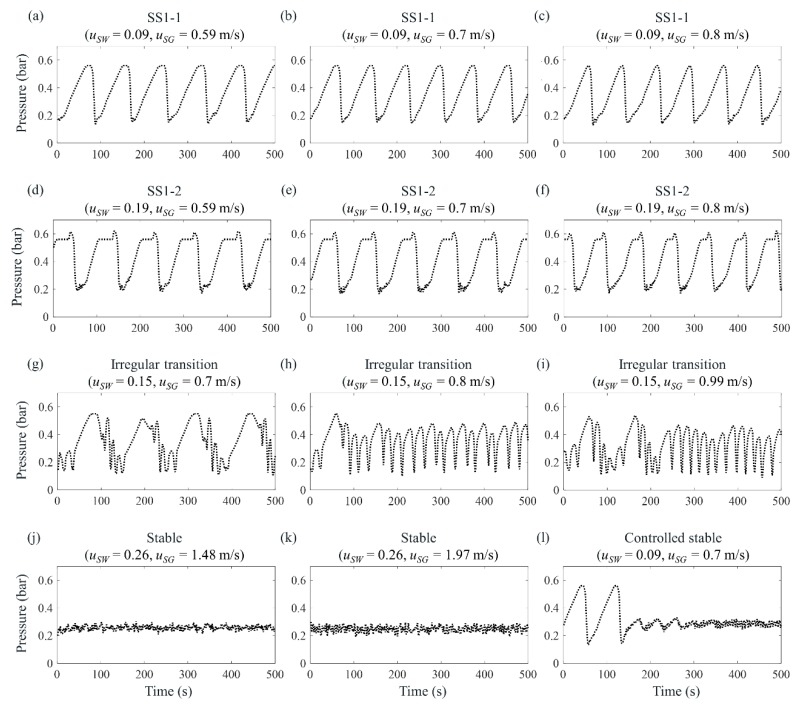
Measured pressure at the riser base for each flow regime: (**a**–**c**) SS1-1 at $u_{SW}$ = 0.09 m/s with $u_{SG}$ = 0.59, 0.7, and 0.8 m/s; (**d**–**f**) SS1-2 at $u_{SW}$ = 0.19 m/s with $u_{SG}$ = 0.59, 0.7, and 0.8 m/s; (**g**–**i**) irregular transition between SS3 and DFSS at $u_{SW}$ = 0.15 m/s with $u_{SG}$ = 0.7, 0.8, and 0.99 m/s; (**j**–**k**) stable flow at $u_{SW}$ = 0.26 m/s with $u_{SG}$ = 1.48 and 1.97 m/s; and (l) controlled stable flow after SS1-1 generation at $u_{SW}$ = 0.09 m/s with $u_{SG}$ = 0.7 m/s.

To generate the different flow regimes of SS1, SS2, transition flow, and stable flow, various conditions of water and gas superficial velocities were tested, of which the number of measured data for each environmental condition is summarized in Table 1. Figure 4 presents the measured pressure at the riser base (*p_B_*) for each flow condition. Three kinds of the first type of SS1, which is called SS1-1 in this paper, occurred under the conditions of $u_{SW}$ = 0.09 m/s with $u_{SG}$ = 0.59, 0.7, and 0.8 m/s, as shown in Figure 4a–c, respectively. When the gas superficial velocity increases, the severe slugging period decreases, because increased gas blocked in the pipeline reduces the time required to exceed the hydrostatic pressure of the accumulated liquid. The second type of SS1, hereinafter referred to as SS1-2, had a longer slug length than that of SS1-1 and three kinds of SS1-2 were generated at $u_{SW}$ = 0.19 m/s with $u_{SG}$ = 0.59, 0.7, and 0.8 m/s as shown in Figure 4d–f, respectively. Because the liquid slug length of SS1-2 was longer than that of SS1-1, it needed more gas compression to blowout. Therefore, higher pressure peaks at the riser base are shown in Figure 4d–f, where these peaks indicate gas blowout. For both SS1-1 and SS1-2, the hydrostatic pressures of the fully accumulated liquid at the riser are 0.56 bar. Next, three types of irregular transition between DFSS and SS3 were tested at $u_{SW}$ = 0.15 m/s with $u_{SG}$ = 0.7, 0.8, and 0.99 m/s, as shown in Figure 4g–i, respectively. In Figure 4i, SS3 is developed prior to approximately 200 s and DFSS is presented later than 200 s, where the high frequency of DFSS is approximately 20 s and the low frequency of DFSS is approximately 100 s. Additionally, two conditions of the stable regime were investigated at $u_{SW}$ = 0.26 m/s with $u_{SG}$ = 1.48 and 1.97 m/s, as shown in Figure 4j,k. *p_B_* ranges from 0.2 to 0.3 bar, which is completely different from that of severe slugging, which fluctuates between 0.2 and 0.6 bar. Lastly, a controlled stable condition was implemented. After SS1-1 was generated at $u_{SW}$ = 0.09 m/s with $u_{SG}$ = 0.7 m/s, a choke valve installed at the top of the riser was operated to eliminate the severe slugging. Choking the valve transforms unstable flow into stable flow by increasing the pressure difference across the choke [5,21] with a proportional-integral-derivative controller. The data measured after flow stabilization were applied to a classification analysis of the stable flow regime. For each severe slugging condition, 1000 data samples were collected, considering that the occurrence interval of severe slugging was approximately 100 s. As a result, approximately 100 cycles of severe slugging were obtained. Meanwhile, 250 samples were acquired for conventional stable flow and 500 samples were measured for controlled stable flow, considering the stabilizing time. Because there was no cyclic process for stable flow, the number of measured data was smaller than that of severe slugging.

**Figure 5:**
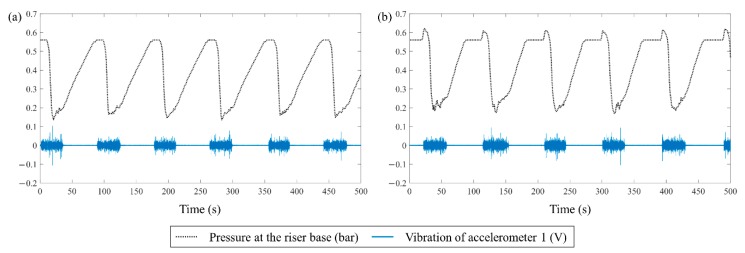
Comparison of the pressure at the riser base and the signals from accelerometer 1 for (**a**) SS1-1 at $u_{SW}$ = 0.09 m/s with $u_{SG}$ = 0.59 m/s and; (**b**) SS1-2 at $u_{SW}$ = 0.19 m/s with $u_{SG}$ = 0.59 m/s. The black dotted line indicates the pressure and the blue solid line indicates the vibration.

Figure 5a,b provide comparisons of *p_B_* and a signal from accelerometer 1 near the riser base during severe slugging cycles of SS1-1 at $u_{SW}$ = 0.09 m/s with $u_{SG}$ = 0.59 m/s and SS1-2 at $u_{SW}$ = 0.19 m/s with $u_{SG}$ = 0.59 m/s, respectively. The black dotted line indicates *p_B_* and the blue solid line shows the vibration signal of accelerometer 1. Although the signals of vibration and pressure are not perfectly synchronized owing to the different data acquisition systems, they present comparable trends of severe slugging. When the liquid starts to accumulate at the riser, which is a starting point of pressure increase, the vibration drastically decreases because an FIV diminishes rapidly. After the blowout, the FIV is developed as the gas penetrates the riser and the pressure starts to decrease. In accordance with the cyclic severe slugging process, the vibration signals also reveal cyclical trend.

**Figure 6:**
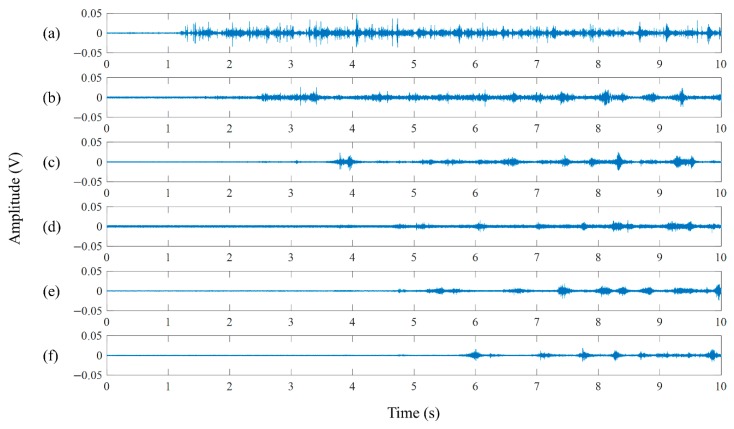
Vibration signals from accelerometers 1–6 in (**a**–**f**), respectively. The flow is generated at $u_{SW}$ = 0.09 m/s with $u_{SG}$ = 0.59 m/s.

**Figure 7:**
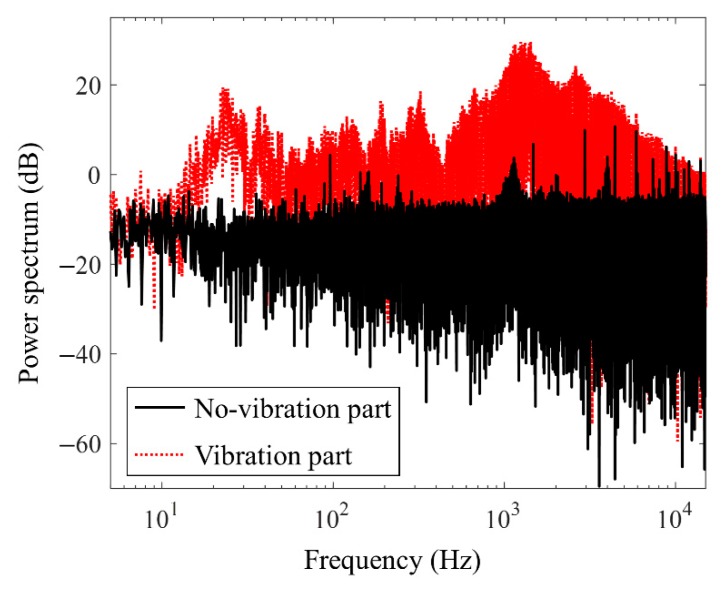
Typical spectrum of FIV. The red dotted line presents the spectrum of FIV and the black solid line is the noise spectrum of the no-vibration part. Most energy of FIV is distributed below 10 kHz, especially near 1.5 kHz and 20 Hz.

Figure 6 illustrates vibration signals of SS1-1 at $u_{SW}$ = 0.09 m/s with $u_{SG}$ = 0.59 m/s near the blowout process, when the FIV begins to be generated. The signals acquired from accelerometers 1–6 are depicted in Figure 6a–f, respectively. The FIV after the blowout is acquired from approximately 1.2 s for accelerometer 1 and 5.6 s for accelerometer 6. The internal two-phase FIV is mainly due to the presence of a flow-turning section such as pipe bend. It changes the momentum flux and pressure fields in a short time, thereby generating large forces [22,23]. Consequently, in the pipeline-riser system of this experiment, the main source of vibration was located at the bottom of the riser, at the turning zone connecting the inclined pipeline and the vertical riser. As shown in Figure 6, the signal amplitude of accelerometer 1 close to the bottom is larger than those of the other sensors. In addition, to investigate the characteristics of the flow noise source, power spectra from the signals of accelerometer 1 about SS1-1 at $u_{SW}$ = 0.09 m/s with $u_{SG}$ = 0.59 m/s are investigated. Figure 7 shows two typical spectra of the no-vibration part during liquid accumulation (black solid line) and the vibration part after the blowout (red dotted line), which are produced using signals of length 10 s. The x-axis represents the frequency on a logarithmic scale and the y-axis shows the power spectrum in decibels. As shown in Figure 7, the energy of the FIV dominates below 10 kHz, especially near 1.5 kHz and 20 Hz.

## 4. Classification Method

### 4.1. Statistic and Linear Prediction Coefficients of Feature Vectors

The vibration signals acquired from the accelerometers provide information about the flow regime, and in particular, the data obtained during the liquid accumulation of the severe slugging process play an important role in the flow recognition. For fast recognition of severe slugging, five simple statistical parameters [24] and linear prediction coefficients (LPCs) are investigated instead of using many types of feature extraction methods such as power spectral density [13] or wavelet denoising [12]. The mean, variance, skewness, kurtosis, root mean square (RMS), and fifth order of LPCs are calculated from the absolute values of measured vibrations x(t)=|s(t)|, where *s*(*t*) is the raw vibration signal. The mean and variance of *x*(*t*) are defined as $F_{1}=E\left[t\right]=\frac{1}{T}\int_{0}^{T}x\left(t\right)dt$ and $F_{2}=E\left[{\left(t-F_{1}\right)}^{2}\right]$, respectively [25], where *T* is a length of signals *x*(*t*). Similarly, the skewness and kurtosis are calculated as:

(1) $$F_{n}=E\left[{\left(\frac{\left(t-F_{1}\right)}{\sqrt{F_{2}}}\right)}^{n}\right]$$

where *n* = 3 and 4 represent the skewness and kurtosis, respectively [26]. The RMS is defined as [27]:

(2) $$F_{5}=\sqrt{\frac{1}{T}\int_{0}^{T}{\left\{x\left(t\right)\right\}}^{2}dt}$$

Meanwhile, linear prediction is a technique to estimate a future value by using previous values. The predicted value is computed by a linear combination of weighted past values x(n−1), x(n−2), …, x(n−p). The linearly predicted value $\hat{x}\left(n\right)$ is defined as [28]:

(3) $$\hat{x}\left(n\right)=-\sum_{k=1}^{p}a_{p}\left(k\right)x\left(n-k\right)$$

where $-a_{p}\left(k\right)$ are weights referred to as LPCs of order *p*. In this study, the order *p* is fixed as 5 because it leads to better classification performance. For 1 ≤ *k* ≤ 5, $-a_{5}\left(k\right)$ are defined as features $F_{i}$(6 ≤ *i* ≤ 10). Ten parameters of feature vectors are listed in Table 2.

### 4.2. Feature Vector Extraction

To classify the flow regime using a short time interval, features were extracted from signals clipped to 7, 5, and 2.5 s in length from the raw data of 10 s length. For the clipped signals of 7 s length, the raw data were divided into two parts of the time intervals [0, 7] and [3, 10], and the data were split into the intervals [0, 5] and [5, 10] for the 5-s-long clipped signals. In the case of the 2.5-s-long clipped signals, the data were clipped as the intervals [0, 2.5] and [2.5, 5], where the last 5 s were not used. The performance of using signals divided into the intervals [0, 2.5], [2.5, 5], [5, 7.5], and [7.5, 10] is quite similar to that of the analysis without using the last 5 s (not shown in this paper). We apply the clipped signals of the intervals [0, 2.5] and [2.5, 5] to balance the size of the feature and the cyclic pattern of severe slugging with the signals of 7 and 5 s.

Ten parameters of a feature vector, including mean, variance, skewness, kurtosis, RMS, and fifth-order LPCs, for each clipped signal were calculated per accelerometer. If the number of accelerometers is *A* and the number of clipped signals is *N*, an input of the learning algorithms becomes an *N* × *M* matrix, where *M* is the dimension of the feature computed by multiplying *A* and the total number of feature parameters 10 in Table 2. For example, if we use six accelerometers with 100 clipped signals, the input feature is a 100 × 60 matrix.

**Figure 8:**
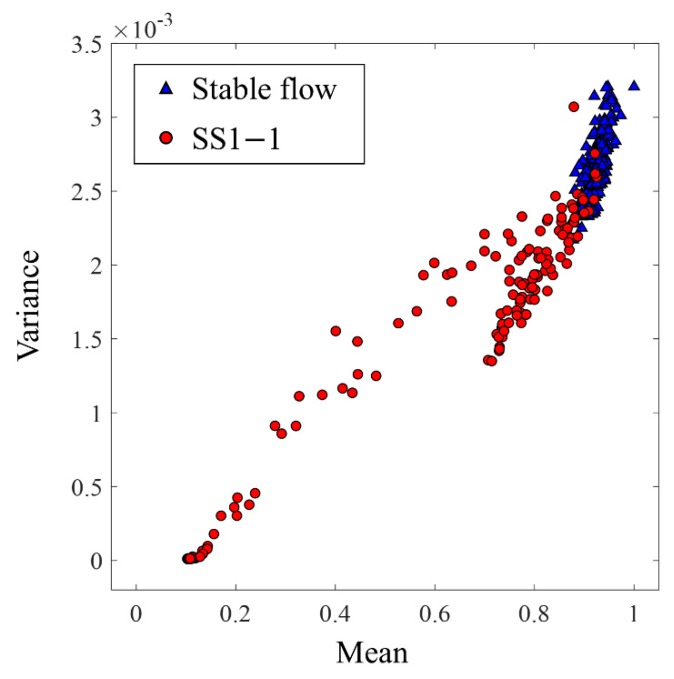
Distribution of mean and variance parameters for stable flow ($u_{SW}$ = 0.26 m/s, $u_{SG}$ = 1.48 m/s) and SS1-1 ($u_{SW}$ = 0.09 m/s, $u_{SG}$ = 0.59 m/s). The red circle markers represent SS1-1 and the blue triangle markers represent stable flow.

Figure 8 illustrates a distribution example of the mean and variance of absolute values of 250 clipped signals in the time interval [0, 7] obtained from accelerometer 1, comparing stable flow at $u_{SW}$ = 0.26 m/s with $u_{SG}$ = 1.48 m/s and SS1-1 at $u_{SW}$ = 0.09 m/s with $u_{SG}$ = 0.59 m/s. The x-axis represents the mean and the y-axis shows the variance, where the maximum value is 1 for the mean parameter because data are normalized. The red circle markers indicate SS1-1 and the blue triangle markers represent stable flow. As shown in Figure 8, the distributions of the two categories are quite separable, in which the values of the mean and variance of stable flow are higher than those of severe slugging because stable flow constantly generates vibration. With respect to the vibration signals of severe slugging, there are two regions according to the cycle of severe slugging: (1) the no-vibration parts during slug formation and slug production (slug movement into the separator) stages, when the FIV diminishes rapidly by single-phase flow with decreasing flow velocity and (2) the vibration parts after the blowout is started, in which the FIV is produced by the momentum flux fluctuation of two-phase flow with increasing flow velocity [23,29,30]. Consequently, the mean and variance of severe slugging are distributed from the lower values at the no-vibration parts to the higher values at the vibration parts near the distribution of stable flow. A significant role of the classifier is to separate the flow regimes carefully in the vicinity of the vibration parts, where the signals for stable flow and severe slugging demonstrate similar patterns.

### 4.3. Binary and Multiclass Classification Method

The supervised learning algorithms of the SVM and NN were adopted to classify the stable flow and severe slugging. The SVM and NN were trained and implemented using the MATLAB statistics and machine learning toolbox and MATLAB neural network tool box, respectively. The SVM is a binary classification algorithm that maximizes a margin given by the smallest distance between the decision hyperplane and any of the data [31]. The SVM finds sparse solutions called support vectors that predict new data with a subset of training data. It solves the optimization problem expressed as [32].

(4) $$\{\begin{array}{c} {}_{}\max_{}\left(\sum_{i=1}^{N}\alpha_{i}-\frac{1}{2}\sum_{i=1}^{N}\sum_{j=1}^{N}\alpha_{i}\alpha_{j}t_{i}t_{j}K\left(y_{i},y_{j}\right)\right)\\ s.t._{}\sum_{i=1}^{N}\alpha_{i}t_{i}=0{,}_{}0\leq \alpha_{i}\leq C{,}_{}i=1,\cdots ,N \end{array}$$

where $y_{i}$ are input vectors; $t_{i}$ are class labels presented as 1 or −1; $\alpha_{i}$ are Lagrange multipliers related to the support vectors; *C* is a parameter determined by the user’s desire, where a larger value of *C* gives a higher penalty to errors; and *K* is a kernel function. A polynomial kernel of degree *p* expressed as $K\left(y_{i},y_{j}\right)={\left(y_{i}\cdot y_{j}+1\right)}^{p}$ or a radial basis function (RBF) $K\left(y_{i},y_{j}\right)=\exp\left(-\frac{{\left\|y_{i}-y_{j}\right\|}^{2}}{2\sigma^{2}}\right)$ is generally used. Then, the decision hyperplane is expressed as:

(5) $$f\left(y\right)=\sum_{i=1}^{N_{S}}\alpha_{i}t_{i}K\left(y_{S},y\right)+b$$

In Equation (5), $y_{S}$ are support vectors, $N_{S}$ is the number of support vectors, and *b* is the bias. By evaluating the sign of f(y) about new data points y, the flow regime can be determined.

As the SVM is generally used as a binary classifier, the other machine learning algorithm NN was applied to both binary and multiclass classifications. The NN is based on $z\left(y,\;w\right)=g\left(\sum_{j=0}^{D}w_{j}\phi_{j}\left(y\right)\right)$ composed of nonlinear basis functions, where g(⋅) is an activation function, $\phi_{j}\left(y\right)$ are nonlinear basis functions, y are input vectors, w present weights, and *D* is the number of units of the layer [31]. For example, the output of the first layer can be expressed as $z_{j}^{\left(1\right)}=\sum_{i=0}^{D}w_{ji}^{\left(1\right)}y_{i}$, where *D* is the number of inputs, the superscript (1) presents the first layer, and *j* = 1, …, *J* (the number of units in the first layer). The outputs of the first layer are transformed by an activation function g(⋅). These quantities are linearly combined again to generate the output of the second layer $z_{l}^{\left(2\right)}=\sum_{j=0}^{J}w_{lj}^{\left(2\right)}g\left(z_{j}^{\left(1\right)}\right)$, which can be expressed as $z_{l}^{\left(2\right)}=\sum_{j=0}^{J}w_{lj}^{\left(2\right)}g\left(\sum_{i=0}^{D}w_{ji}^{\left(1\right)}y_{i}\right)$, *l* = 1, …, *L* (the number of units in the second layer). The hidden layer can be constructed successively in the same manner. In a recent study of NNs, the rectifier linear unit defined as $g\left(z\right)=\max\left\{0{,}_{}z\right\}$ was recommended as the activation function [33], where *z* are activations to be transformed by the activation function [31], and applied in this study. The size of the input layer is *M* (*A* × 10) and the output layer has two neurons for stable flow and severe slugging.

**Figure 9:**
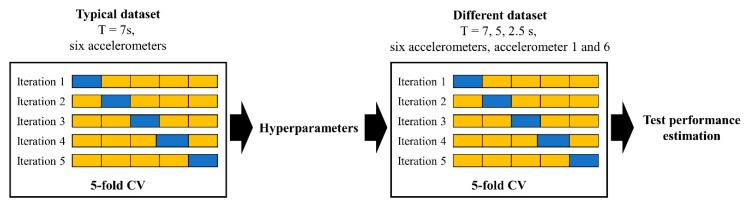
The process of 5-fold CV and estimating the test performance. The 5-fold CV performance is computed by averaging the 5 estimates of the test results. Based on the optimized hyperparameters using the typical dataset composed of 7-s-long signals with six accelerometers, the test performances are evaluated and compared with different test datasets.

Meanwhile, hyperparameters for the SVM and NN such as the kernel function, parameters of the kernel function, layers, and nodes were optimized to maximize the 5-fold cross-validation (CV) performances of using typical dataset composed of 7-s-long signals with six accelerometers. In general, when a large assigned test set is not available to compute the test performance, the *k*-fold CV performance can be used to estimate the performance [34]. For binary classification analysis, the number of samples selected from each of the nine severe slugging conditions must be controlled in order to balance the number of samples between the stable flow class and severe slugging class. Within a limited number of samples, the information about the severe slugging could be imbalanced according to the cyclic process of the severe slugging, which implies that the performance could be affected by the assigned training, validation, and test set. Therefore, the 5-fold CV is adopted to estimate the generalized test performance. Figure 9 illustrates the process of 5-fold CV and demonstrates the procedure of evaluating the performance based on the 5-fold CV. First, the total dataset is partitioned into 5 groups (folds) of equal size. When the first fold is applied as a validation set, the model trains on the remaining 4 folds. This process is repeated 5 times, where each different 4 group of dataset is treated as a validation set. Then, the 5-fold results are generated after 5 iterations, and the performance is estimated by mean CV accuracy. After fixing the optimized hyperparameters using the typical dataset composed of 7-s-long signals with six accelerometers, the test performances are evaluated and compared with different datasets.

Prior to computing feature parameters for binary classification, the total datasets were constructed from various flow conditions. For the stable flow class, 225 original samples were chosen from each of two stable conditions at $u_{SW}$ = 0.26 m/s with $u_{SG}$ = 1.48 and 1.97 m/s. After the original samples were clipped into two parts such as [0, 5] and [5, 10] for 5-s-long signals, the data samples from two stable conditions were doubled to 900 samples. Accordingly, total 900 clipped samples for the stable flow class were constructed, which are computed as 225 (original samples from each stable condition) × 2 (flow conditions) × 2 (clipping process). With the 900 clipped samples, 10 feature parameters of Table 2 are calculated to produce feature vectors of the stable flow class. Similarly, the feature vectors of the severe slugging class were constructed for nine conditions of the severe slugging as listed in Table 1. In performance evaluation and comparison using machine learning algorithms, it is important to balance the number of data for each class. Therefore, 50 original samples from each of the nine severe slugging conditions were selected sequentially to produce a total of 900 clipped samples for the severe slugging class, calculated as 50 (original samples from each severe slugging condition) × 9 (flow conditions) × 2 (clipping process).

## 5. Classification Results and Discussion

### 5.1. Binary Classification Results

In this study, three cases of adopting accelerometers are investigated using: (1) all six accelerometers; (2) accelerometer 1 at the riser base; and (3) accelerometer 6 at the top of the riser. Therefore, the dimension of the feature *M* is 60 or 10, according to the number of accelerometers. The purpose of the analysis applying accelerometer 1 and 6 is to examine the effect of sensor location and the possibility of adopting a small number of sensors. Based on the typical dataset (validation set) composed of 7-s-long signals with six accelerometers, hyperparameters were optimized. The averaged accuracies for the typical dataset are 99.78% for the SVM, and 100% for the NN.

Table 3 presents the binary classification results of the SVM and NN. The input is a 900 × 60 matrix for six accelerometers and a 900 × 10 matrix for one accelerometer. The averaged accuracy, the accuracy for stable flow, and that for severe slugging are demonstrated according to the signal length based on the accelerometer selection. Because long time signals have more information about the flow regime, they result in better performance than those of short time signals. Hence, using 7-s-long signals with six accelerometers yields the best performance, where the averaged accuracies are approximately 99.67% for the SVM and 99.72% for the NN. When the signal length decreases to 2.5 s, the averaged accuracies also degenerate to 96.5% for the SVM and 97.89% for the NN. Although the NN needs more training time, it yields slightly better averaged accuracies than the SVM. Furthermore, the accuracies for stable flow are higher than those for severe slugging. This is because a main factor distinguishing between the two classes is the no-vibration parts during the liquid accumulation of severe slugging, whereas identifying the vibration parts between severe slugging and stable flow is more complicated. Therefore, the misclassification probability of severe slugging increases in the vicinity of the vibration parts, as shown in Figure 8. Moreover, the sensor location can affect the performance because the major source of FIV is located at the bottom of the riser. In Table 3, the signals from accelerometer 1, close to the riser base, lead to better performance than the signals from accelerometer 6, near the top of the riser, because of the clearer distinctions between the vibration parts and no-vibration parts.

In the aforementioned results, only 50 samples were extracted out of 1,000 data for each severe slugging condition to balance the size of the features with the stable flow class. To investigate the performance difference according to sample group selection in the severe slugging data, additional analysis is performed on clipped signals of length 7 s obtained by using all six accelerometers through the SVM. The results are presented in Table 4, where the first column indicates the group number of the samples of severe slugging. For each sample group, a different sequence of 50 samples was extracted from each severe slugging condition, and the data of the stable class were the same as in Table 3. For example, the first 50 samples were selected as group 1, the next 50 samples were chosen as group 2, and the next 50 samples were used as the subsequent group. This was repeated 10 times to check a total of 500 samples. The averaged accuracies of different groups are similar, which have a maximum of 99.67% and a minimum of 97.83%. This is because each group of samples are in time series composed of approximately 4–6 cycles of severe slugging. Therefore, the sample group selection cannot significantly affect the classification performance by similar cyclic patterns.

Finally, the effect of the controlled stable condition was tested by converting the features of stable flow to include three stable flow conditions, as listed in Table 1. One hundred and fifty samples from each stable flow conditions were selected and clipped according to signal length. The number of data for the stable class is 900, calculated as 150 (samples from each stable condition) × 3 (flow conditions) × 2 (clipping process), and the features of severe slugging are same as in Table 3. Table 5 presents the classification results of the SVM and NN. The averaged accuracies are comparable to those of the previous analyses in Table 3. The FIV of the controlled stable condition presents less continuous patterns than the conventional stable flow, which is associated with the valve opening. In most cases, it leads to higher accuracies of severe slugging and lower accuracies of stable flow than those of the previous analyses in Table 3, except for signals of length 5 and 2.5 s with six accelerometers using the NN. In these two cases, the accuracies for severe slugging are lower than those in Table 3 by 0.34% and 2%. Similarly, the accuracies degenerate as the signal length decreases.

In the recent study of Zou et al. [14], the shortest signals of 6.8 s were tested based on the trained LS-SVM using 8.99-s-long signals. The averaged accuracy was 93.78%, where the accuracy of unstable flow was 94.9% and that of stable flow was 90.57%. Apart from the machine learning algorithm, there are some differences from this study: (1) the data were acquired from three types of differential pressures of the riser; (2) the number of each class for binary classification was different; (3) the unstable flow regime composition was different, which had SS1, SS2, SS3, OSC, and unstable cycles of an irregular region. Although direct comparison is difficult because of the aforementioned differences, it is worth mentioning that our results obtained with shorter signals show comparable performance to their results. Overall, this presents some advantages of accelerometer signals and satisfactory performance is achieved in binary classification; in particular, the performance obtained by using 2.5-s-long signals shows the possibility of real-time monitoring.

### 5.2. Multiclass Classification Results

The main objective of multiclass classification using the NN is to recognize stable flow, SS1-1, SS1-2, and the irregular transition between SS3 and DFSS. The features of the stable class cover two stable flow conditions at $u_{SW}$ = 0.26 m/s with $u_{SG}$ = 1.48 and 1.97 m/s. 240 samples were extracted from each stable flow condition and doubled by a clipping process. Accordingly, 960 data in total were computed as 240 (samples from each stable condition) × 2 (flow conditions) × 2 (clipping process). In the case of SS1-1, 160 samples were extracted from three conditions of $u_{SW}$ = 0.09 m/s with $u_{SG}$ = 0.59, 0.7, and 0.8 m/s. A total of 960 data were produced by 160 (samples from each severe slugging condition) × 3 (flow conditions) × 2 (clipping process), and the processes of data generation of SS1-2 and irregular transition are same as that of SS1-1. Based on the typical dataset (validation set) composed of 7-s-long signals with six accelerometers, hyperparameters were optimized. The averaged accuracy for the typical dataset is 97.89%, where that of the stable flow, SS1-1, SS1-2, and irregular transition are 100%, 96.98%, 97.6%, and 96.98%, respectively. Table 6 presents the multiclass classification results with six accelerometers using the NN. The averaged accuracies range from 94.9% to 97.06% according to the signal length. The accuracies for stable flow are higher than those of severe slugging, which results from the vibration parts after the blowout of each severe slugging being comparable to each other. This also renders the averaged accuracies of multiclass classification slightly lower than those of binary classification.

## 6. Summary and Conclusions

In this paper, the severe slugging recognition in a pipeline-riser system using signals from accelerometers was proposed. Vibrations were obtained from the bottom to the top of the vertical riser under various environmental conditions generated by different water and gas superficial velocities. For online recognition of the flow regime, the simple features of mean, variance, skewness, kurtosis, RMS, and fifth-order LPCs were extracted from the absolute values of the vibration signals. The SVM and NN were used for binary classification and the NN was adopted in multiclass classification to recognize stable flow, SS1-1, SS1-2, and an irregular transition between SS3 and DFSS. The hyperparameters were optimized to minimize the five-fold cross-validation errors.

Our classifiers show the best performance in signals of length 7 s with six accelerometers. Although the performance degenerates for short time signals, the averaged accuracy of binary classification of 2.5-s-long signals with six accelerometers is quite high, at 97.89%, using the NN. The recognition rate of stable flow tends to be higher than that of severe slugging, because separating the vibration parts of severe slugging and stable flow is more intricate. When the number of accelerometers decreases to one, the accuracy of accelerometer 1 near the riser base is higher than that of accelerometer 6 close to the top of the riser. This is because the major source of FIV is located at the bottom of the riser, the flow-turning section of the structure. As a result, signals from the accelerometer near the riser base have a clearer distinction between the vibration and no-vibration parts, thereby yielding better classification performance. The averaged accuracies for multiclass classification using six accelerometers range from 94.9% to 97.06% according to the signal length. As identifying each severe slugging is more complicated owing to their similar tendencies of the vibration parts after the blowout, the recognition rates of multiclass classification are slightly lower than those of binary classification.

The success of early recognition of severe slugging is based on the measuring device and simple features. Our results show that the use of signals from accelerometers leads to good performance, as vibration measurements are a great discriminator in the no-vibration parts during the liquid accumulation state of the severe slugging cycle. Based on our analysis, adding information from accelerometer signals can further improve the accuracy and reliability of established severe slugging monitoring systems.

## Figures and Tables

**Table 1:** Water and gas superficial velocities and the number of measured data according to the flow regime.

Flow Regime	Water Superficial Velocity (m/s)	Gas Superficial Velocity (m/s)	Number of Measured Data
SS1-1	0.09	0.59	1000
		0.7	1000
		0.8	1000
SS1-2	0.19	0.59	1000
		0.7	1000
		0.8	1000
Transition between SS3 and DFSS	0.15	0.7	1000
		0.8	1000
		0.99	1000
Stable	0.26	1.48	250
		1.97	250
Controlled stable	0.09	0.7	500

**Table 2:** Summary of feature parameters. *x*(t) is defined as *x*(t) = |*s*(*t*)| and −*a*_i_(*i* − 5) are the LPCs of Equation (3).

Feature		Equation
*F*_1_	Mean	$F_{1}=E\left[t\right]=\frac{1}{T}\int_{0}^{T}x\left(t\right)dt$
*F*_2_	Variance	*E* [${\left(t-F_{1}\right)}^{2}$]
*F*_3_	Skewness	*E* [${\left(\left(t-F_{1}\right)/\sqrt{F_{2}}\right)}^{3}$]
*F*_4_	Kurtosis	*E* [${\left(\left(t-F_{1}\right)/\sqrt{F_{2}}\right)}^{4}$]
*F*_5_	RMS	$\sqrt{\int_{0}^{T}{\left\{x\left(t\right)\right\}}^{2}dt/T}$
*F_i_* (6 ≤ *i* ≤ 10)	LPC	−*a*_5_(*i* − 5)

**Table 3:** Binary classification results of the SVM and NN.

Accelerometer	Length of Signals (s)	Averaged Accuracy (%) SVM/NN	Accuracy for Stable Flow (%) SVM/NN	Accuracy for Severe Slugging (%) SVM/NN
1–6	7	99.67/99.72	99.78/100	99.56/99.44
	5	98.83/98.94	99.22/99.22	98.44/98.67
	2.5	96.5/97.89	97.56/97.67	95.44/98.11
1	7	93.17/95.22	98.78/97.22	87.56/93.22
	5	91.28/93.17	97.67/95.56	84.89/90.78
	2.5	89.67/89.89	97.33/93.89	82/85.89
6	7	86/86.67	97.67/93.44	74.33/79.89
	5	84.94/84.17	97.67/92	72.22/76.33
	2.5	81.94/82.44	97.44/91.11	66.44/73.78

**Table 4:** Binary classification results of the SVM for signals of length 7 s with six accelerometers.

Data Group	Averaged Accuracy (%)
1	97.83
2	98.11
3	98.28
4	98.5
5	99.06
6	99.39
7	99.44
8	99.28
9	99.06
10	99.67
Maximum	99.67
Minimum	97.83
Average	98.86

**Table 5:** Binary classification results of the SVM and NN including controlled stable regime.

Accelerometer	Length of Signals (s)	Averaged Accuracy (%) SVM/NN	Accuracy for Stable Flow (%) SVM/NN	Accuracy for Severe Slugging (%) SVM/NN
1–6	7	99.67/99.56	99.78/99.33	99.56/99.78
	5	98.89/99	99/99.67	98.78/98.33
	2.5	96.72/96.94	97.11/97.78	96.33/96.11
1	7	93.83/95.5	98.33/97.11	89.33/93.89
	5	92.22/94.28	96.78/95.56	87.67/93
	2.5	88.89/90.33	93.56/91.89	84.22/88.78
6	7	87.06/87.5	95.33/91.67	78.78/83.33
	5	85.44/86	95.88/91.22	75/80.78
	2.5	82.78/82.22	93/87.33	72.56/77.11

**Table 6:** Multiclass classification results of the NN with six accelerometers.

Length of Signals (s)	Averaged Accuracy (%)	Accuracy for Stable Flow (%)	Accuracy for SS1-1 (%)	Accuracy for SS1-2 (%)	Accuracy for Irregular Transition (%)
7	97.06	99.79	95.83	96.35	96.25
5	97.03	99.58	95.63	96.46	96.46
2.5	94.9	98.44	93.65	94.38	93.13

## References

[1] Schmidt Z., Doty D.R., Dutta-Roy K. (1985). Severe slugging in offshore pipeline riser-pipe systems. Soc. Pet. Eng. J..

[2] Yocum B.T. Offshore riser slug flow avoidance: mathematical models for design and optimization. Proceedings of the SPE European Meeting.

[3] Hill T.J., Wood D.G. Slug flow: occurrence, consequences, and prediction. Proceedings of the University of Tulsa Centennial Petroleum Engineering Symposium.

[4] Kang C., Wilkens R., Jepson W.P. The effect of slug frequency on corrosion in high pressure, inclined pipelines. Proceedings of the CORROSION 96, NACE International.

[5] Pedersen S., Durdevic P., Yang A. (2017). Challenges in slug modeling and control for offshore oil and gas productions: A review study. Int. J. Multiph. Flow.

[6] Sun J.Y., Jepson W.P. Slug flow characteristics and their effect on corrosion rates in horizontal oil and gas pipelines. Proceedings of the SPE Annual Technical Conference and Exhibition.

[7] Blaney S., Yeung H. (2008). Investigation of the exploitation of a fast-sampling single gamma densitometer and pattern recognition to resolve the superficial phase velocities and liquid phase water cut of vertically upward multiphase flows. Flow Meas. Instrum..

[8] Goudinakis G. (2004). Investigation on the Use of Raw Time Series and Artificial Neural Networks for Flow Pattern Identification in Pipelines. Ph.D. Thesis.

[9] Mi Y., Ishii M., Tsoukalas L.H. (1998). Vertical two-phase flow identification using advanced instrumentation and neural networks. Nucl. Eng. Des..

[10] Rosa E.S., Salgado R.M., Ohishi T., Mastelari N. (2010). Performance comparison of artificial neural networks and expert systems applied to flow pattern identification in vertical ascendant gas–liquid flows. Int. J. Multiph. Flow.

[11] Trafalis T.B., Oladunni O., Papavassiliou D.V. (2005). Two-phase flow regime identification with a multiclassification support vector machine (SVM) model. Ind. Eng. Chem. Res..

[12] Wu H., Zhou F., Wu Y. (2001). Intelligent identification system of flow regime of oil–gas–water multiphase flow. Int. J. Multiph. Flow.

[13] Ye J., Guo L. (2013). Multiphase flow pattern recognition in pipeline–riser system by statistical feature clustering of pressure fluctuations. Chem. Eng. Sci..

[14] Zou S., Guo L., Xie C. (2017). Fast recognition of global flow regime in pipeline-riser system by spatial correlation of differential pressures. Int. J. Multiph. Flow.

[15] Hernandez L., Juliá J.E., Chiva S., Paranjape S., Ishii M. (2006). Fast classification of two-phase flow regimes based on conductivity signals and artificial neural networks. Meas. Sci. Technol..

[16] Juliá J.E., Liu Y., Paranjape S., Ishii M. (2008). Upward vertical two-phase flow local flow regime identification using neural network techniques. Nucl. Eng. Des..

[17] Schmidt Z., Brill J.P., Beggs H.D. (1980). Experimental study of severe slugging in a two-phase-flow pipeline-riser pipe system. Soc. Pet. Eng. J..

[18] Taitel Y. (1986). Stability of severe slugging. Int. J. Multiph. Flow.

[19] Baliño J.L., Burr K.P., Nemoto R.H. (2010). Modeling and simulation of severe slugging in air–water pipeline–riser systems. Int. J. Multiph. Flow.

[20] Malekzadeh R., Henkes R.A.W.M., Mudde R.F. (2012). Severe slugging in a long pipeline–riser system: experiments and predictions. Int. J. Multiph. Flow.

[21] Farghaly M.A. Study of severe slugging in real offshore pipeline riser pipe system. Proceedings of the 5th Society of Petroleum Engineers Middle East Oil Show.

[22] Cargnelutti M.F., Belfroid S.P., Schiferli W. (2010). Two-phase flow-induced forces on bends in small scale tubes. J. Press. Vessel Technol..

[23] Miwa S., Mori M., Hibiki T. (2015). Two-phase flow induced vibration in piping systems. Prog. Nucl. Energy.

[24] Jung S., Seong W., Lee K. (2018). Damage detection on an aluminum plate from the cross-correlation of diffuse field using the support vector machine. Ocean Eng..

[25] Bendat J.S., Piersol A.G. (2011). Random Data: Analysis and Measurement Procedures.

[26] Gubner J.A. (2006). Probability and Random Processes for Electrical and Computer Engineers.

[27] Zill D.G., Wright W.S., Cullen M.R. (2006). Advanced Engineering Mathematics.

[28] Proakis J.G., Manolakis D.G. (1996). Digital Signal Processing: Principles, Algorithms and Applications.

[29] Liu Y., Miwa S., Hibiki T., Ishii M., Morita H., Kondoh Y., Tanimoto K. (2012). Experimental study of internal two-phase flow induced fluctuating force on a 90° elbow. Chem. Eng. Sci..

[30] Riverin J.L., Pettigrew M.J. (2007). Vibration excitation forces due to two-phase flow in piping elements. J. Press. Vessel Tech..

[31] Bishop C. (2006). Pattern Recognition and Machine Learning.

[32] Burges C.J. (1998). A tutorial on support vector machines for pattern recognition. Data Min. Knowl. Discov..

[33] Goodfellow I., Bengio Y., Courville A. (2016). Deep Learning.

[34] James G., Witten D., Hastie T., Tibshirani R. (2013). An Introduction to Statistical Learning.

